# Myeloperoxidase gene knockout causes local inflammation and dysbiosis in the murine gut

**DOI:** 10.1080/29933935.2025.2548210

**Published:** 2025-08-28

**Authors:** Jack J. Wang, Yawen Hu, Scott Jennings, Meng Luo, Christopher M. Taylor, William M. Nauseef, Guoshun Wang

**Affiliations:** a Department of Microbiology, Immunology and Parasitology, School of Medicine, Louisiana State University Health Sciences Center, New Orleans, LA, USA; b Inflammation Program, Roy J. and Lucille Carver College of Medicine, University of Iowa, and Veterans Administration Medical Center, Iowa City, IA, USA

**Keywords:** Myeloperoxidase, gut microbiota, intestinal inflammation, bowel movement, epithelial integrity

## Abstract

Myeloperoxidase (MPO), predominantly expressed in neutrophils, catalyzes the production of hypochlorous acid (HOCl) that plays an integral role in the host defense against invading pathogens. However, little is known about its role in maintaining normal gut microbiome and function. Here, we report the comparisons of inflammation and microbiome in the intestines of WT and MPO^–/–^ mice. Immune cell profiling demonstrated that the MPO^−/−^ mice had significantly more neutrophils and T cells in their intestinal mucosae and significantly more fecal calprotectin as compared to the WT mice. Fluorescent dextran permeability of the bowel showed no difference between the two strains of mice. Carmine Red transit demonstrated that the MPO^−/−^ intestines had slower movements than did the WT controls. Sequencing the intestinal 16S rDNA of co-housed MPO^−/−^and WT mice identified 13 bacterial families, 2 of which were unique to MPO^−/−^ and 7 to WT mice. Alpha diversity of the microbiome in WT intestines was significantly higher than that of MPO^−/−^ ones, and beta diversity of the two microbial communities of the two genotypes also differed significantly. Functional pathway analyses revealed distinct metabolic signatures. Thus, normal MPO function is important to intestinal health and its deficiency leads to gut inflammation and dysbiosis.

## Introduciton

Polymorphonuclear leukocytes (PMNs) or neutrophils employ a variety of antimicrobial agents to support their vital role in the host defense against infection.^1,2^ Prominent among the microbicidal responses is the generation of oxidants through the action of the phagocyte NADPH oxidase and the delivery of proteins by fusion of intracellular granules with phagosomes.^3^ Hydrogen peroxide (H_2_O_2_) produced by the phagocyte NADPH oxidase reacts with myeloperoxidase (MPO),^4^ a protein present at high concentrations in azurophilic granules of PMNs, to generate Compound I, which in turn catalyzes the two-electron oxidation of chloride anion (Cl^−^) to hypochlorous acid (HOCl), a potent microbicide.^5,6^

Individuals with inherited defects in oxidant-dependent responses underscore the clinical importance of the MPO-H_2_O_2_-Cl^−^ system. PMNs from individuals who lack a functional phagocyte oxidase or MPO fail to kill microbes normally.^7^ Specifically, patients with a defective NADPH oxidase develop chronic granulomatous disease (CGD), which leads to recurrent infections, predominantly by *Nocardia*, *Aspergillus* species, and catalase-positive bacteria including *Staphylococcus aureus* and *Burkholderia cepacia*.^8–10^ Patients with MPO deficiency exhibit a less dramatic phenotype than do patients with CGD, but frequently experience severe infections with *Candida albicans*, particularly in the presence of concomitant diabetes mellitus.^11^

Whereas the importance of the oxidant-dependent system to PMN antimicrobial activities is well established, the contribution of this system to the health of gut microbiome and function is incompletely characterized. To address this knowledge gap, we employed a murine model of MPO deficiency to investigate how the MPO-H_2_O_2_-Cl^−^ system influences intestinal inflammatory status, gut motility, and the composition and diversity of the intestinal microbiome. The obtained data suggest that MPO in PMNs plays a vital role in regulating intestinal inflammation, supporting a healthy gut microbiome, and ensuring normal bowel function.

## Materials and methods

### Animals and ethics of animal use

B6.129X1-Mpo^tm1Lus^/J (MPO-knockout or MPO^−/−^) mice and WT control mice were originally purchased from the Jackson Laboratory and bred in the corresponding author’s (GW) laboratory. In the MPO^−/−^ mice, *Mpo* Exon 7 was disrupted by the insertion of a neomycin selection cassette and a premature stop codon. Three genotyping primers were used: 1) Common forward 5’-AGGTCTCTAACGCCATCGTG-3’; 2) WT reverse 5’-GTTGAGGCCAGTGAA GAAGG-3’; 3) Mutant reverse 5’-CACGAGACTAGTGAGACGTG-3.’ All animals used in this study were under C57BL/6J background and were age- and sex-matched, ranging from 4 to 12 weeks old. The animals were handled according to the ethical standards set by the Guide for Care and Use of Laboratory Animals of the National Institutes of Health and approved by the Louisiana State University Health Sciences Center Animal Care and Use Committee.

### Animal co-housing, intestinal tissue collection and microbial DNA extraction

MPO^−/−^ and WT (MPO^+/+^) mice at 4–5 weeks old were co-housed for 2 months to normalize for potential environmental influences on gut microbiota. For tissue collection, each mouse was euthanized by CO_2_ asphyxiation and the intestine dissected out and placed in cold phosphate buffered saline (PBS). Next, the entire intestine with contents from each mouse was homogenized with 3–5 power cycle strokes at maximal speed using a sterile electronic homogenizer. The QIAamp DNA Microbiome kit (Qiagen, Valencia, CA) was used for isolation of the microbiota DNAs. This kit allowed us to enzymatically deplete host cell DNA prior to bacterial lysis. A modification was made by combining mechanical and chemical cell disruptions to reduce the potential bias introduced by the differential susceptibility of bacterial cell walls to a particular lysis procedure. The purified and enriched bacterial DNAs were applied for 16S rRNA gene-based microbiome analysis.

### Enzyme linked immunosorbent assay (ELISA)

MPO^−/−^ and WT mice were individually placed in single containers, and their fresh fecal pellets were collected and snap-frozen in liquid nitrogen and stored at −80°C until further processed. The pellets from each mouse were then weighed, thawed and mashed in ice-cold PBS containing Roche protease inhibitor cocktail (Millipore-Sigma). The suspensions were centrifuged at a high speed for 15 min at 4°C, and the supernatants collected and subjected to ELISA. The Mouse S100A8/S100A9 Heterodimer DuoSet ELISA kit (R&D system, Minneapolis, MN) and MPO DuoSet ELISA kit (R&D system, Minneapolis, MN) were used to measure the levels of fecal calprotectin and MPO according to the manufacturers’ instructions.

### Intestinal mucosal cell isolation, immunostaining and flow cytometry

Small intestines of MPO^−/−^ and WT mice were excised at the pyloric sphincter and the ileocecal valve. The tissues were separately placed in ice-cold PBS (Gibco), longitudinally cut open, and transversely severed into ~1 cm segments. Next, the tissues were repeatedly washed in cold PBS with vigorous shaking to cleanse the contents. Then, the clean tissues were placed in HBSS with 1 mM EDTA (Gibco) and 1 mM DTT (Sigma) and incubated at 37°C with shaking for 20 min. After sedimentation, the supernatants were collected. Then, the tissues were placed in RPMI 1640 with 1 μM collagenase (Sigma) and 1 μM DNase (Sigma) and digested at 37°C for 15 min with shaking. This brief digestion largely dissociated mucosal cells, including intestinal epithelial cells and resident immune cells. Then, the digestion supernatants were collected, correspondingly combined with the first collected supernatants, and filtered through a 70 μm strainer. The isolated mucosal cells (1×10^6^) were spun down and resuspended in PBS with 1% BSA (Fisher Bioreagents) and Fc blocker (BD Biosciences CD16/CD32 Purified Rat Anti-Mouse) for 10 min, and immunostained using the listed antibody panel (**Supplementary data, Table S1**) on ice in the dark for 40 min. After washing, the stained cells were fixed in 2% paraformaldehyde (Fisher Bioreagents). Differentials of the intestinal mucosal cells were assessed using flow cytometry (BD FACSymphony A3) and analyzed using FlowJo v10.8.0.

### Intestinal epithelial permeability assay

Mice were fasted for 4 h before receiving an oral gavage of 0.6 mg/g FITC-Dextran (MW: 3,000–5,000; Millipore-Sigma). Blood samples were collected from each mouse before gavage, as well as 2 and 4 h after gavage. Sera obtained were diluted in PBS at a 1:4 ratio, and the fluorescence intensity of each sample was measured using a BioTek Cytation 5 Cell Imaging Multimode Reader (Agilent) with excitation/emission wavelengths of 485 nm/528 nm. A non-relevant mouse serum was spiked with dose-escalating FITC-Dextran to establish a standard curve for calculation of the absolute concentration of the FITC-Dextran in each serum.

### Gut transit assay

Oral gavage solution was made by dissolving 6% (w/v) Carmine Red (Sigma-Aldrich) and 1.5% (w/v) methylcellulose (Sigma-Aldrich) in hot autoclaved mouse drinking water. Mice were weighed and gavaged with the solution at a volume of 1% of body weight through a 21 G blunt-end feeding needle. The mice were then placed in single containers. Dropping of fecal pellets was monitored every 10 min. Transit time was recorded from T0 (the time of gavage) to the time of first appearance of a completely red pellet.

### 16S rRNA gene sequencing

The 16S ribosomal DNA segment containing the hypervariable V3 and V4 regions was amplified using 20 ng of isolated genomic DNA and gene-specific primers with Illumina adaptors: Forward 5’-TCGTCGGCAGCGTCAGATGTGTATAAGAGACA GCCTACGGGAGGCAGCAG-3’; Reverse 5’-GTCTCGTGGGCTCGGAGATGTGTATAAGAGACAGGGACTACHVGGGTWTCTAAT-3’. The PCR condition was as follows: 95°C for 3 min; 25 cycles of 95°C for 30 s, 55°C for 30 s and 72°C for 30 s; 72°C for 5 min; and holding at 4°C. The PCR product from each mouse was purified using AMPure XP beads added as 0.85x PCR volume. The purified amplicon DNA (2 μl) from the last step was amplified eight cycles under the same PCR condition using primers with different molecular barcodes: forward 5’-AATGATACGGCGACCACCGAGATCTACAC [i5] TCGTCGGCAGCGTC-3’; reverse 5’-CAAGCAGAAGACGGCATACGAGAT [i7] GTCTCGTGGGCTCGG-3’. The indexed amplicon libraries purified using AMPure XP beads and quantified using Quant-iT PicoGreen (Invitrogen) were normalized and pooled. The pooled library was quantified using the KAPA Library Quantification Kit (Kapa Biosystems), diluted and denatured as the guideline of Illumina’s sequencing library preparation. 10% PhiX was added to the sequencing library as an internal control and to increase diversity of the 16S RNA amplicon library. The paired-end sequencing was performed on an Illumina MiSeq (Illumina, San Diego, CA) using the 2 × 250bp V2 sequencing kit.

### 16S rRNA gene sequencing data analyses

The obtained 16S rRNA gene sequences were analyzed using Qiime 2 v2023.9.^12^ Raw FASTQ data, formatted in Casava 1.8 paired-end demultiplexed fastq, were imported into Qiime2 and subjected to quality control using the q2-dada2 plugin. To effectively align V3 and V4 sequencing fragments and trim low-quality reads, the dada2 denoise-paired method was employed with the following settings: “–p-trim-left-f 20 –p-trim-left-r 20 –p-trunc-len-f 251 –p-trunc-len-r 190.” Following quality control, a substantial number of paired reads were retained for each sample for subsequent analyses (**Supplementary data, Table S2**).

Alpha- (α-) and beta (β-) diversity analyses were carried out utilizing the q2-diversity plugin. Taxonomic analysis was performed using the q2-feature-classifier plugin and Silva-138–99-nb-classifier (**Supplementary data, Table S3**). Differential abundance (DA) of taxonomic composition at different levels across the samples were calculated using the ANalysis of COmpositions of Microbiomes (ANCOM) method via q2-composition plugin (**Supplementary data, Table S4**). All generated data were visualized in Qiime2 View.

Analyses of MetaCyc Metabolic Pathway and Kyoto Encyclopedia of Genes and Genomics (KEGG) Pathway were conducted using the q2-picrust2 plugin in Qiime2 (**Supplementary data, Table S5 and S6**). Conversions of absolute abundance to relative abundance were performed using the relabund() function within the R package: IgAScores (version 0.1.2).^13^ Further analysis and visualization were performed in an R package: ggpicrust2 (version 1.7.3).^14^ For differential abundance analysis of pathways, the Linear models for differential abundance analysis of microbiome compositional (LinDA) method was employed. P-values were adjusted using the Benjamini-Hochberg correction method. Top differentially abundant pathways were defined as those with a p.adjusted value of <0.05 and a log_2_ (average abundance fold change) >1.

### Statistical analyses

Two-group comparisons were conducted using a two-tailed, unpaired Student’s t-test. The data were presented as mean ± standard deviation (SD). A significance with *p* < 0.05 was considered statistically different. Statistical analyses and graphical representations were performed using GraphPad Prism 10 or R programming (version 4.3.1).

## Results

### Mice with MPO gene knockout exhibit intestinal inflammation

**Figure 1.**
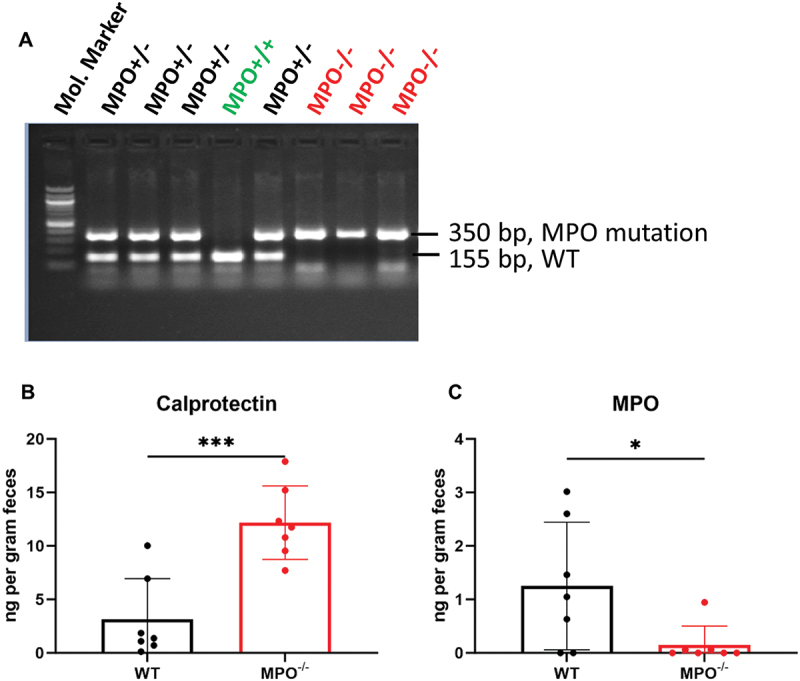
Confirmation of MPO mutation in MPO^−/−^ mice. (A) a representative genotyping PCR shows the insertion mutation in mouse MPO gene. (B&C) ELISA results show expression levels of calprotectin and MPO in the fecal pellet of WT and MPO^−/−^ mice. Data were presented as mean ± SD. A 2-tailed, unpaired Student’s t-test was used to determine statistic differences (*n* = 7, *p < 0.05, ***p < 0.001).

To create congenic MPO^−/−^ and MPO^+/+^ (WT) mice, we crossbred the parental MPO^−/−^ mice (B6.129X1-Mpo^tm1Lus^/J) with WT C57BL/6J mice to produce MPO^+/−^ heterozygotes first. Then, the MPO^+/−^ mice were interbred to generate both wild-type (MPO^+/+^) and MPO knockout (MPO^−/−^) mice for experiments, shown in a representative genotyping Figure 1A. Clinically, calprotectin and MPO are two diagnostic markers used to assess intestinal inflammation. As they are expressed predominantly in neutrophils, elevated levels in the intestine are considered as strong indicators of neutrophil infiltration and degranulation, key features signifying the presence of active inflammation. To determine if the absence of MPO in PMNs affects intestinal inflammation, we measured the levels of fecal calprotectin and MPO of WT and MPO^−/−^ mice. MPO^−/−^ mice had significantly higher levels of fecal calprotectin than did WT mice (Figure 1B), suggesting that the intestines of MPO^−/−^ mice were inflamed. Because MPO^−/−^ mice fail to produce MPO, no fecal MPO was detectable in the MPO^−/−^ mice (Figure 1C), validating the MPO gene knockout.

**Figure 2.**
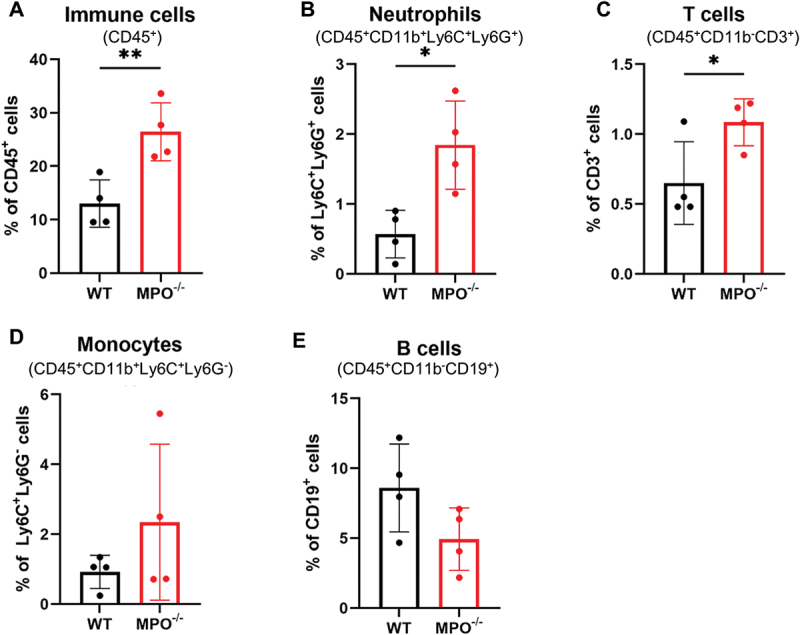
Differential of immune cells isolated from intestinal lamina propria shows neutrophilic inflammation in MPO^−/−^ mice. (A) Percentage of immune cells (CD45^+^) in all live lamina propria cells. (B) Percentage of neutrophils (CD11b^+^Ly6C^+^Ly6G^+^) in CD45^+^ cells. (C) Percentage of T cells (CD11b^−^CD3^+^) in CD45^+^ cells. (D) Percentage of monocytes (CD11b^+^Ly6C^+^Ly6G^−^) in CD45^+^ cells. (E) Percentage of B cells (CD11b^−^CD19^+^) in CD45^+^ cells. Data were presented as mean ± SD. A 2-tailed, unpaired Student’s t-test was used to determine statistic differences (*n* = 4, *p < 0.05, **p < 0.01, ***p < 0.001).

Inflammation is a physiological response to tissue injury or microbial infection that typically progresses stepwise, from initiation to resolution, each phase with a characteristic cellular profile.^15^ To assess intestinal inflammation in MPO^−/−^ mice, we profiled the immune cells isolated from the mucosae of their small intestines. Flow cytometry using the established gating strategy (**Supplementary data, Fig. S1**) demonstrated that the percentages of CD45^+^ marrow-derived immune cells and Ly6C^+^Ly6G^+^ PMNs were significantly higher in the MPO^−/−^ intestines than in the WT controls (Figrue 2A,B), suggesting that the inflammation in MPO^−/−^ mice was neutrophilic, although there were also significantly more CD3^+^ T cells in the MPO^−/−^ intestine (Figure 2C). In contrast, the relative infiltrations of Ly6C^+^Ly6G^−^ monocytes and CD19^+^ B cells in the two strains were not different (Figure 2D,E).

### MPO gene knockout affects intestinal transit rate but not permeability

**Figure 3.**
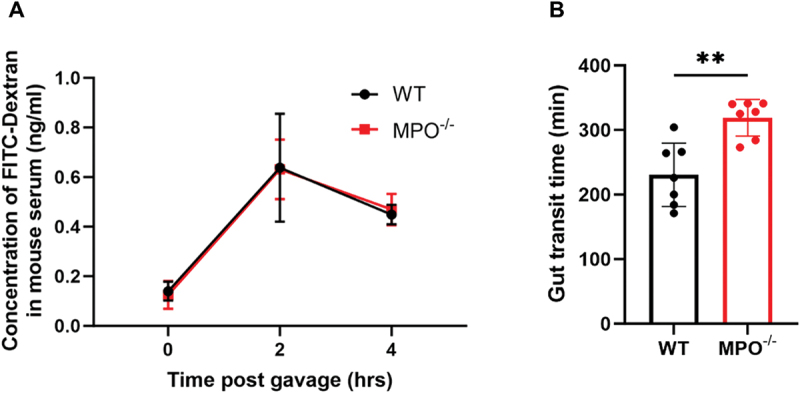
MPO loss of function does not affect intestinal permeability, but slows bowel movement. (A) Concentration of FITC-Dextran in mouse serum. WT and MPO^−/−^ mice were evaluated for intestinal permeability at 0, 2, and 4-hour post-FITC-Dextran gavage. *n* = 8. (B) Carmine Red dye passing shows significant difference in fecal transit time. WT and MPO^−/−^ mice were measured for gut transit from the time of Carmine Red gavage (T0) to the time of the first completely red fecal pellet. Statistical differences were determined by unpaired Student’s t-test (*n* = 7, **p < 0.01).

Local inflammation may interfere with intestinal epithelial integrity and function. To determine if the absence of MPO from PMNs altered intestinal permeability and mobility, we measured the serum concentrations of FITC-Dextran before and after oral gavage of the fluorescence. MPO^−/−^ and WT mice had similar intestinal permeability at the tested time points (Figure 3A), indicating that MPO loss of function did not affect the epithelial tightness of the intestines. To measure the rate of bowel movement, we measured the transit of the non-absorbable Carmine Red dye after oral gavage. In WT mice, the average transit time of the dye was 231 min, whereas MPO^−/−^ mice took a significantly longer time (312 min) (Figure 3B), suggesting that the bowel movement was delayed in the absence of MPO.

### MPO gene knockout alters gut microbiota

**Figure 4.**
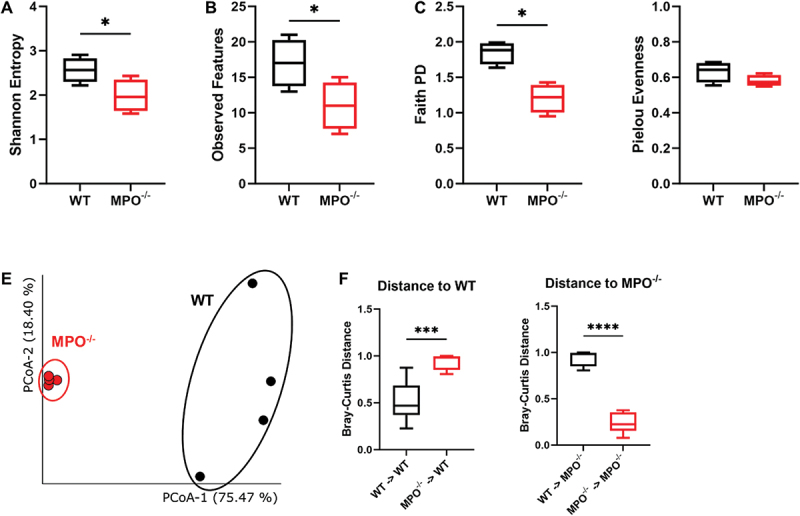
Bacterial community diversity in the WT and MPO^−/−^ gut. (A-D) alpha diversity measured by different methods: Shannon Entropy (A), observed features (B), faith PD (C), and pielou evenness (D). Significant differences were determined by Kruskal-Wallis pairwise test (*p < 0.05). (E&F) beta diversity measured by Bray-Curtis method. (E) Principal coordinate analysis (PCoA) of the microbial community based on Bray-Curtis distance matrix. (F) the Bray-Curtis distance among samples intra- and inter-groups. Significant differences were determined by a 2-tailed, unpaired Student’s t-test (***p < 0.001, ****p < 0.0001).

Given the recognized importance of PMNs in mucosal immunity^16^ and our data showing MPO^−/−^ mice had increased neutrophilic inflammation and delayed bowel transit, we hypothesized that the intestinal microbiota would be altered in MPO^−/−^ mice. To test this hypothesis, we employed 16S rRNA sequencing of intestinal flora from MPO^−/−^ and WT to compare their intestinal microbiota, judging differences in the overall microbial community by α- and β-diversity analyses. Metrics of α-diversity, indicative of the richness of a microbial community within each sample, demonstrated significantly reduced values of Shannon Entropy, Observed Features, and Faith PD in the microbiota from the MPO^−/−^ intestines as compared to those from the WT controls (Figure 4A-C). However, microbial evenness was similar in MPO^−/−^ and WT mice (Figure 4D). Taken together, these results suggest that the richness of the commensal bacteria in MPO^−/−^ intestines was diminished as compared to that in WT intestines. Moreover, β-diversity, indicative of dissimilarity of bacterial communities across different groups, revealed a great deviation of the gut microbiome in MPO^−/−^ mice from that in WT mice (Figure 4E). The distances between the individual samples displayed significant disparities in bacterial composition within the intestines of WT and MPO^−/−^ mice (Figure 4F). Similar results were also obtained using other qualitative methods of β diversity analysis (**Supplementary data, Fig. S2**).

**Figure 5.**
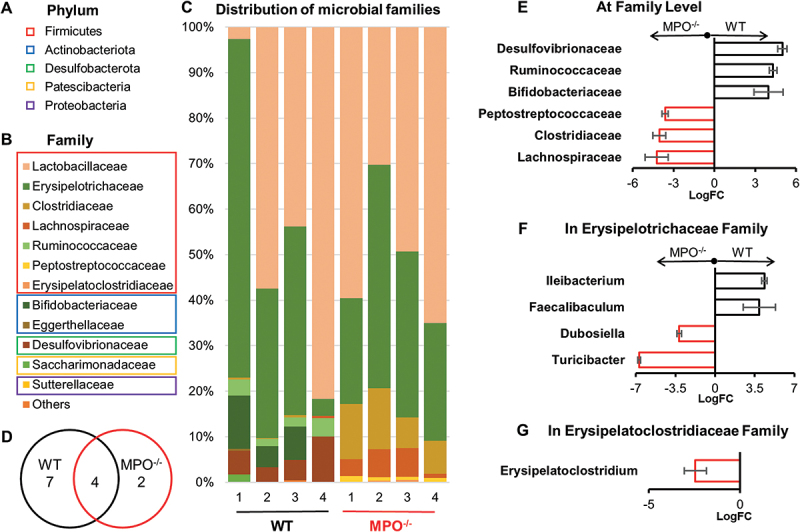
Taxonomic composition and bacteria differential abundance in the WT and MPO^−/−^ gut. (A-C) The bacterial compositions in the intestinal content of WT and MPO^−/−^ mice. (D) Venn diagram showing the number of bacterial family in the intestinal microbiota of WT and MPO^−/−^ mice. (E-G) Differential abundance (DA) analysis of bacteria between WT and MPO^−/−^ groups. (E) Differently abundant bacteria in WT and MPO^−/−^ mice at family level. (F) Differently abundant bacteria in WT and MPO^−/−^ mice at genus level in the family of *Erysipelotrichaceae*. (G) Differently abundant bacteria in WT and MPO^−/−^ mice at genus level in the family of *Erysipelatoclostridiaceae*. The analyses were performed by Qiime2 plugin with ANCOM method, all significantly differently abundant bacteria were defined by *p* < 0.05 and q < 0.05. Full report of DA analysis of intestinal microbiota between WT and MPO^−/−^ were displayed in Supplementary data, Table S4. FC, fold-change.

At the phylum level, the MPO^−/−^ intestinal microbiome was composed solely by Firmicutes, whereas the WT harbored a more diverse microbiota, including Firmicutes, Antinobacterota, Desulfobacterota, Patescibacteria, and Proteobacteria Figure 5A-C. At the family level, out of a combined total of 13 bacterial families, 11 were identified in WT mice, seven of which were exclusive to this strain. In contrast, the intestinal microbiome of MPO^−/−^ mice had six families, only two of which were unique to the knockout strain (Figure 5D). These differences are consistent with the lower α-diversity observed in MPO^−/−^ mice (Figure 4A-C). Differential Abundance (DA) analysis revealed (Figure 5(E-G)) that the dominant bacterial families in the WT intestines were *Desulfovibrionaceae*, *Ruminococcaceae*, and *Bifodobacterianceae*, whereas *Peptostreptococcaceae*, *Clostridiaceae*, and *Lachnosporaceae* were dominant in the MPO^−/−^ intestines (Figure 5E). Moreover, *Erysipelotrichaceae* and *Erysipelatoclostridiaceae* did not show significant DA values at the family level. However, within the *Erysipelotrichaceae* family, *Ileibacterium* and *Faecalibaculum* were differentially abundant in WT mice, whereas *Dunosiella* and *Turicibacter* were differentially abundant in MPO^−/−^ mice at the genus level (Figure 5F). Additionally, *Erysipelatoclostridium* in the *Erysipelatoclostridiaceae* family exhibited differential abundance in MPO^−/−^ mice (Figure 5G). These findings indicate that dissimilar microbiomes existed in the WT and MPO^−/−^ intestines. Taken together, all these microbiota data revealed that MPO loss of function is correlated with a marked reduction in bacterial community richness within the intestines and a significant alteration in microbial composition.

### Different predicted functions of intestinal microbiota from MPO^−/−^and WT mice

**Figure 6.**
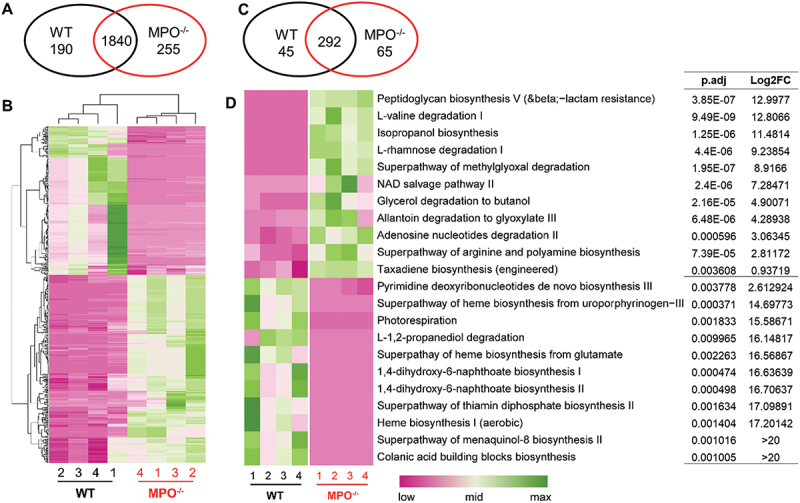
MetaCyc pathway analysis and prediction of gut microbiome characteristics in the WT and MPO^−/−^ gut. (A&B) Differential abundance (DA) analysis of enzyme classification (E.C.) numbers between WT and MPO^−/−^ groups. (A) Venn diagram showing the number of differentially abundant E.C. numbers in each group. (B) Heatmap showing the differentially abundant KOs in the two groups. (C&D) DA analysis of predicted MetaCyc pathways between WT and MPO^−/−^ groups. (C) Venn diagram showing the number of differentially abundant MetaCyc pathways in each group. (D) Heatmap showing the differentially abundant MetaCyc pathways in the two groups. The statistical analyses were performed by ggpicrust2 package with LinDA methods with a benjamin-hochberg correction. Adjusted *p* value < 0.05 were considered as significant differences. Full report of MetaCyc pathway analysis between WT and MPO^−/−^ were documented in Supplementary data, Table S5 and S6. FC, fold-change.

Specific pathway analysis of a microbiome can predict its metabolic signature, such as its ability to produce certain metabolites, degrade specific compounds or induce special host cell responses. To this end, we performed MetaCyc Metabolic Pathway and Kyoto Encyclopedia of Genes and Genomics (KEGG) pathway analyses, using PICRUSt 2.^17^ In total, we detected 2,285 enzymes/molecules with Enzyme Commission (E.C.) numbers associated with our sequencing data. Of these, 190 were significantly more abundant in the microbiome of WT mice, whereas 255 were more abundant in the intestines of MPO^−/−^ mice (Figure 6A,B). These E.C. numbers were used to calculate MetaCyc pathway abundances and coverages.^17^ Out of 402 predicted MetaCyc pathways, 45 were highly abundant in WT mice and 65 in MPO^−/−^ mice (Figure 6C). We highlighted the top five and other prominent pathways from each group (Figure 6D). Many pathways for the degradation of amino acids and small molecules (e.g., L-valine, L-rhamnose, methylglyoxal, glycerol, allantoin, adenosine nucleotides) were predicted forMPO^−/−^ mice, whereas pathways involved in synthesis of many essential molecules and metabolites were associated with the microbiome in WT mice (Figure 6D). These findings suggest that the presence of MPO might favor intestinal microbiota to adopt a biochemical profile skewed toward small molecule synthesis and productive metabolic activities.

**Figure 7.**
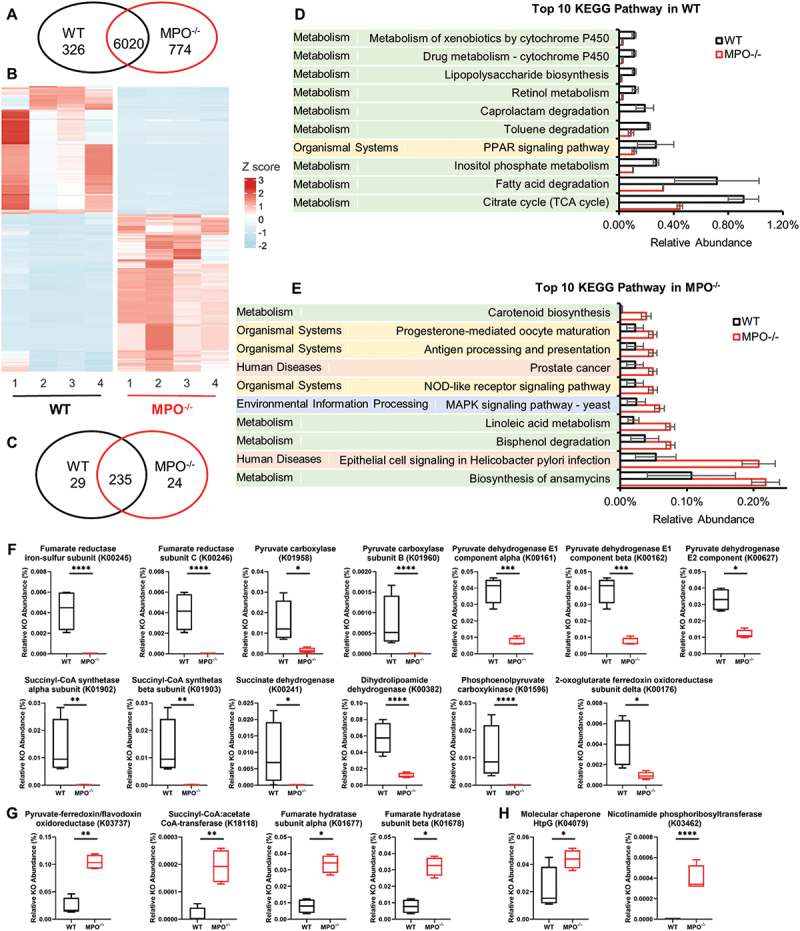
KEGG pathway analysis and prediction of gut microbiome characteristics in the WT and MPO^−/−^ gut. (A&B) Differential abundance (DA) analysis of KEGG orthologies (KOs) between WT and MPO^−/−^ groups. (A) Venn diagram showing the number of differentially abundant KOs in each group. (B) Heatmap showing the differentially abundant KOs in the two groups. (C-E) DA analysis of predicted KEGG pathways between WT and MPO^−/−^ groups. (C) Venn diagram showing the number of differentially abundant KEGG pathways in each group. (D) Top ten differential abundant KEGG pathways in WT group. (E) Top ten differential abundant KEGG pathways in MPO^−/−^ group. (F) 13 differential abundant KOs enriched in citrate cycle (TCA cycle) in the WT group. (G) 4 differential abundant KOs enriched in citrate cycle (TCA cycle) in the MPO^−/−^ group. (H) 2 differential abundant KOs enriched in NOD-like receptor signaling pathway in the MPO^−/−^ group. The statistical analyses were performed by ggpicrust2 package with LinDA methods with a Benjamin-Hochberg correction. Adjusted *p* value < 0.05 were considered as significant differences (*p.adj < 0.05, **p.adj < 0.01, ***p.adj < 0.001, ****p.adj < 0.0001). Full report of KEGG pathway analysis between WT and MPO^−/−^ were documented in Supplementary data, Table S5 and S6.

KEGG pathway analysis provides a broader perspective on biological processes. Among the total of 7120 KEGG Orthologies (KOs) obtained, 326 KOs were significantly abundant in WT mice, whereas more than twice as many KOs, 774, were significantly abundant in MPO^−/−^ mice (Figure 7A, B). As each KO represents a pivotal connecting point in a KEGG pathway, enrichment of the KEGG pathway can be judged by multiple related KOs. A Venn diagram analysis demonstrated that 29 KEGG pathways were significantly abundant in WT mice, whereas 24 KEGG pathways were abundant in MPO^−/−^ mice (Figure 7C). Of the 29 pathways in WT, 26 (89.7%) were associated with metabolism, whereas the remaining 3 were related to organismal systems, genetic information processing, and human diseases. In contrast, of the 24 pathways in MPO^−/−^ mice, 50% pertained to metabolism, whereas the other pathways belonged to organismal systems (16.7%), environmental information processing (8.3%), human diseases (8.3%), cellular processes (8.3%), and genetic information processing (8.3%).

The top ten differentially abundant KEGG pathways in each genotype were examined, which reflected the most functional differences of the gut microbiota (Figure 7D, E). For example, citrate cycle (TCA cycle), which serves as the hub of aerobic metabolism in microbes, exhibited significant alterations. In WT mice, 13 significantly abundant KOs were involved in the TCA cycle pathway, encompassing enzymes such as fumarate reductase, pyruvate carboxylase, pyruvate dehydrogenase, and others (Figure 7F). Conversely, only four KOs involved in the TCA cycle were significantly more abundant in MPO^−/−^ mice (Figure 7G). In the MPO^−/−^ group, two KOs were significantly altered (molecular chaperone HtpG and nicotinamide phosphoribosyltransferase), whereas none was observed in WT mice (Figure 7H). Collectively, these data suggest that multiple pathways may be altered by the dysregulated microbiome and thereby impose adverse effects on the host.

## Discussion

The mucosal surface of gastrointestinal tract serves as an extensive interface between the environment and the body and consequently needs to deflect microbial invasion from without to maintain homeostasis within. To that end, epithelial cells and their secretions, along with both resident and circulating immune cells, collaborate to maintain tissue integrity. As the predominant immune cell in circulation, PMNs contribute to optimal mucosal immunity, as underscored by the occurrence of inflammatory diseases of the bowel that are frequently associated with inherited neutrophil defects.^18^ For example, individuals with CGD, a genetic defect in any one of the components of the phagocyte NADPH oxidase that compromises the ability of stimulated neutrophils to generate oxidants, incur frequent and often fatal infection.^8^ As many as 40–50% of CGD patients develop symptoms and signs suggestive of inflammatory bowel disease (IBD).^19^ Furthermore, optimal oxidant-dependent antimicrobial activity of human PMNs relies on the action of the granule protein myeloperoxidase to generate hypochlorous acid (HOCl), a potent microbicide.^5^ Balanced redox signaling in the local mucosa dictates the status of inflammation in the gastrointestinal tract.^20^ Our data indicate that MPO^−/−^ mice had intestinal mucosal inflammation with excessive infiltration of PMNs and T cells (Figure 2). In addition, we detected significantly more calprotectin, a neutrophil-associated protein, in the feces of MPO^−/−^ mice (Figure 1), suggesting that more neutrophils were not only recruited to the intestinal mucosa but also into the intestinal lumen. While the underlying mechanism governing this phenotype requires further study, it is likely that the compromised antimicrobial activity of MPO-deficient neutrophils^7,21^ undermined successful killing and elimination of local microbial invaders, resulting in unresolved infection and sustained stimulation of local epithelium, which triggers overproduction of neutrophil chemoattractants, chemokines or leukotriene B4.^22^ Thus, MPO-deficient mice had more PMNs recruited to the submucosal area and subsequently migrated into the lumen, promoting neutrophilic inflammation in the intestine.

In addition to its modified inflammatory status, the MPO-deficient bowel exhibited statistically significantly slower intestinal transit (Figure 3B), although epithelial cell integrity in the bowel was intact in the absence of MPO (Figure 3A). Prolonged gastrointestinal transit time is a prominent feature of many inflammatory bowel conditions, including severe ulcerative colitis,^23^ and cystic fibrosis.^24,25^ As gut motility involves the coordination of the nervous, gastrointestinal, and musculoskeletal systems,^26^ the mechanism behind the retarded movement in MPO^−/−^ mice likely involves multiple factors. However, inflammation is apparently a key trigger to this condition. One possibility is that intestinal inflammation activates inducible nitric oxide synthase to overproduce nitric oxide that causes intestinal relaxation and slows its movement.^27^

Gut microbiota is a complex microbial ecosystem that co-evolved with the host to form a reciprocal and mutually beneficial symbiotic relationship. For example, the host immune system restricts and fine-tunes the gut microbiota whereas the microbiome contributes to the induction, education, and function of the host immune system. Together, their reciprocal activities ensure intestinal health.^28,29^ Our data clearly demonstrated that MPO gene knockout significantly altered the gut microbiota. We also found that the intestinal flora of MPO^−/−^ mice had decreased alpha diversity and that Firmicutes had become the dominant type of microbes (Figures 3,4), indicating the presence of an aberrant gut microbiome. Firmicutes encompasses more than 200 different genera, such as *Lactobacillus*, *Bacillus*, *Clostridium*, *Enterococcus*, and *Ruminicoccus*, among which *Clostridium* represents 95% in the human gut.^30^ Members from this phylum can be beneficial or detrimental to host health. An imbalance in the Firmicutes/Bacteroidetes ratio is reported to link to conditions like obesity, IBD, and metabolic dysfunction.^31^ An increase in Firmicutes has been observed in conditions like rheumatoid arthritis.^32^ To date, no gut microbiota data are published on people with MPO deficiency. However, MPO-deficient mice show a substantial microbiome shift with dextran sulfate sodium (DSS) induction of colitis.^33^ Moreover, CGD patients, who lack oxidant production in neutrophils and other phagocytes, have significantly reduced alpha diversity and increased relative abundance of Proteobacteria.^34^ In addition, our beta diversity data showed distinct clustering of microbiome between the MPO^−/−^ and WT mice (Figure 4), which likewise parallels differences seen between the gut microbiomes of CGD patients and healthy cohorts. Thus, neutrophil oxidant production is involved in shaping the intestinal microbiome, and defects of such function can cause dysbiosis, manifesting as loss of microbiome diversity and dominance of a few microbes. How a neutrophil functional alteration influences the intestinal microbiome remains poorly defined. We believe that neutrophils, continuously mobilized to the mucosal surface, constantly phagocytose and eliminate microbes, which select the permissive bacteria to colonize the mucosal surface and to subsequently seed the intestinal contents for amplification. When neutrophils are defective in oxidant production, some bacteria that should otherwise be eliminated thrive and populate the intestines.

Our MetaCyc pathway analysis indicates that the microbes in the MPO^−/−^ intestines had significantly higher abundance in pathways that degrade key amino acids and small molecules, whereas these microbes in the WT intestines had higher abundance of biosynthetic pathways (Figure 6). In consistent with this finding, an analysis of stool samples from CGD patients reveals significant abundances in pathways such as isopropanol biosynthesis, NAD salvage, allantoin degradation, propanediol degradation, and glycerol degradation.^35^ These alterations in the metabolic profiles of the microbiomes from CGD patients resemble those seen in our MPO knockout mice and thus suggest that similar oxidant-dependent events lead to similar alterations in metabolic pathways and pathogenic mechanisms in both settings.

Taken together, our findings expand the discovery that neutrophil-mediated oxidant production impacts gut microbiota and function to include the products of MPO-dependent reactions. The role of neutrophil as sentinels of the gut mucosa is well-recognized,^16^ and defects in neutrophil function alter the inflammatory state and microflora in the gut.^18^ There is compelling evidence that MPO^−/−^ mice exhibit aberrant immune responses beyond those directly due to neutrophil activity, including both exaggerated as well as muted inflammatory responses.^35–37^ When studied in experimental models of colitis, MPO^−/−^ mice show less inflammation and more rapid resolution^38^ or no protection^33^ in comparison to the responses of WT mice. What is clear from our studies is that the inflammatory state and microbiome in the intestines of MPO^−/−^ mice differs from those in WT mice at baseline, in the absence of any chemical or infectious challenge. Thus, contingent on the disease setting or condition, MPO can be pro- or anti-inflammation. Furthermore, our data on immune profile changes within the MPO^−/−^ intestines contrast with the published report.^33^ This inconsistency may be derived from different mouse strains, and/or different microbiome profiles. We also found the infiltration of T-cells in the mucosa of MPO knockout mice, suggesting the involvement of the acquired immunity in response to the abnormal intestinal microbiome that sculpt the inflammation. Hence, complex and reciprocal interactions between the intestinal commensals and elements of the entire immune system, particularly those dependent on neutrophil function, underlie our observations in the MPO^−/−^ mice.

This study, which utilized the MPO-knockout mouse model, has inherent limitations, as murine findings may not be directly translatable to human physiology. Therefore, future investigation of the gut microbiota and function of MPO-deficient humans is warranted. If our mouse data are validated in humans, restoration of neutrophil function may be developed into a viable therapeutic strategy to correct the dysbiosis and to restore the gut normal function.

In summary, our data demonstrated that MPO-null mice displayed an abnormal inflammatory response, delayed bowel transit, and intestinal dysbiosis. These findings suggest that MPO-dependent events, presumably the products of the MPO-H_2_O_2_-Cl^−^ system in neutrophils, strongly influence intestinal mucosal immunity, the intestinal microbiome, and, consequently, gut health.

## Supplementary Material

Supplementary Table S2.xlsx

Supplementary Table S4.xlsx

Figure S1.TIF

Supplementary Table S3.xlsx

Supplementary Table S5.xlsx

Original Image for Fig 1A.tif

Figure S2.TIF

Wang et al 2025_Supplementary Information.docx

Supplementary Table S6.xlsx

## Data Availability

The metagenomics data from this research have been deposited to the Sequence Read Archive (SRA) of the National Center for Biotechnology Information (NCBI) with a BioProject number (PRJNA1246994). The codes used for our analysis are available upon request.
