# Supplementary figures and images for: Myeloperoxidase gene knockout causes local inflammation and dysbiosis in the murine gut
Source: Gut Microbes Rep. 2025 Aug 28;2(1):2548210. doi: 10.1080/29933935.2025.2548210 (PMC12700598; doi:10.1080/29933935.2025.2548210)

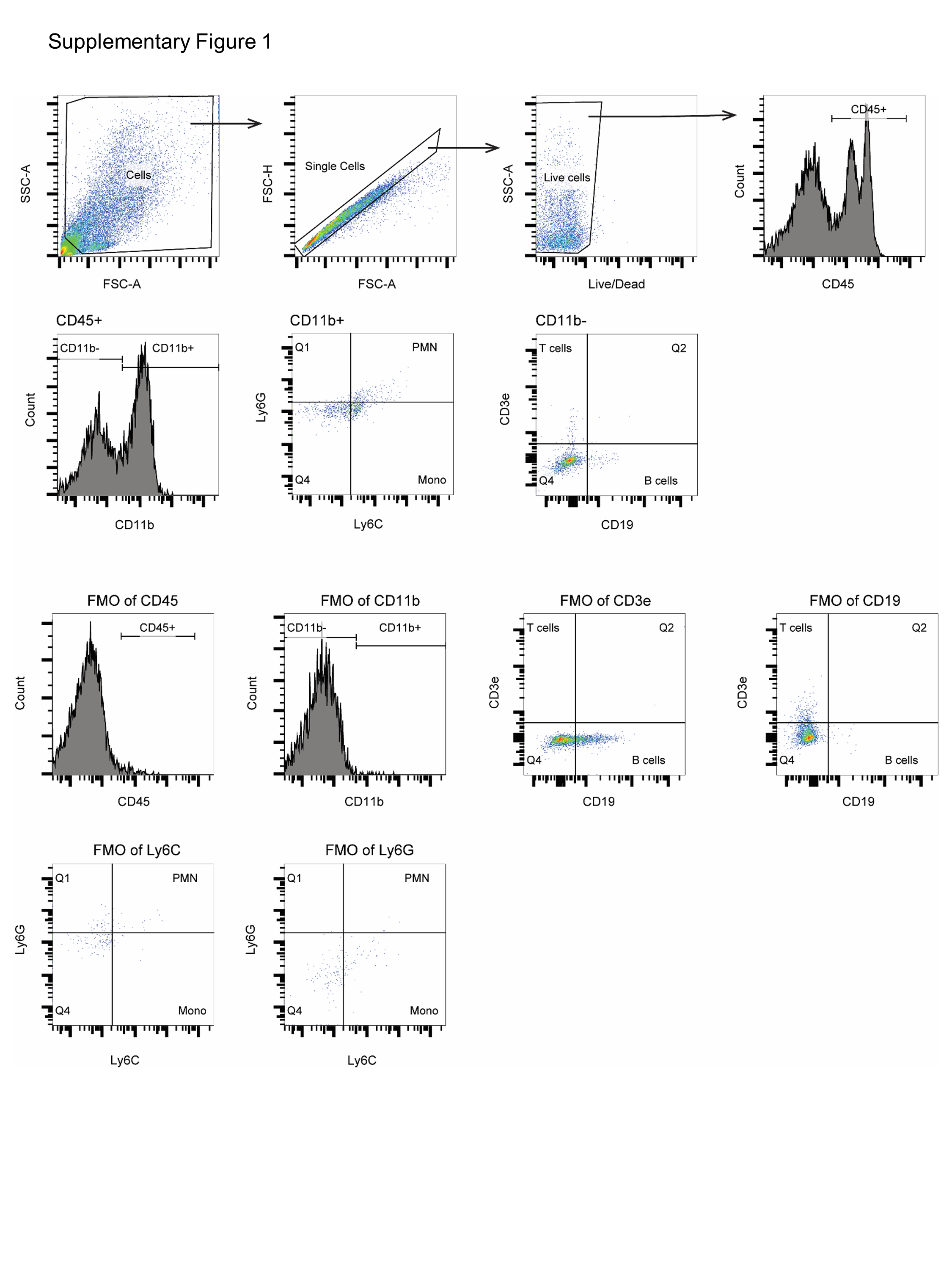

Supplement: Figure S1.TIF [file KGMR_A_2548210_SM4053.tif]

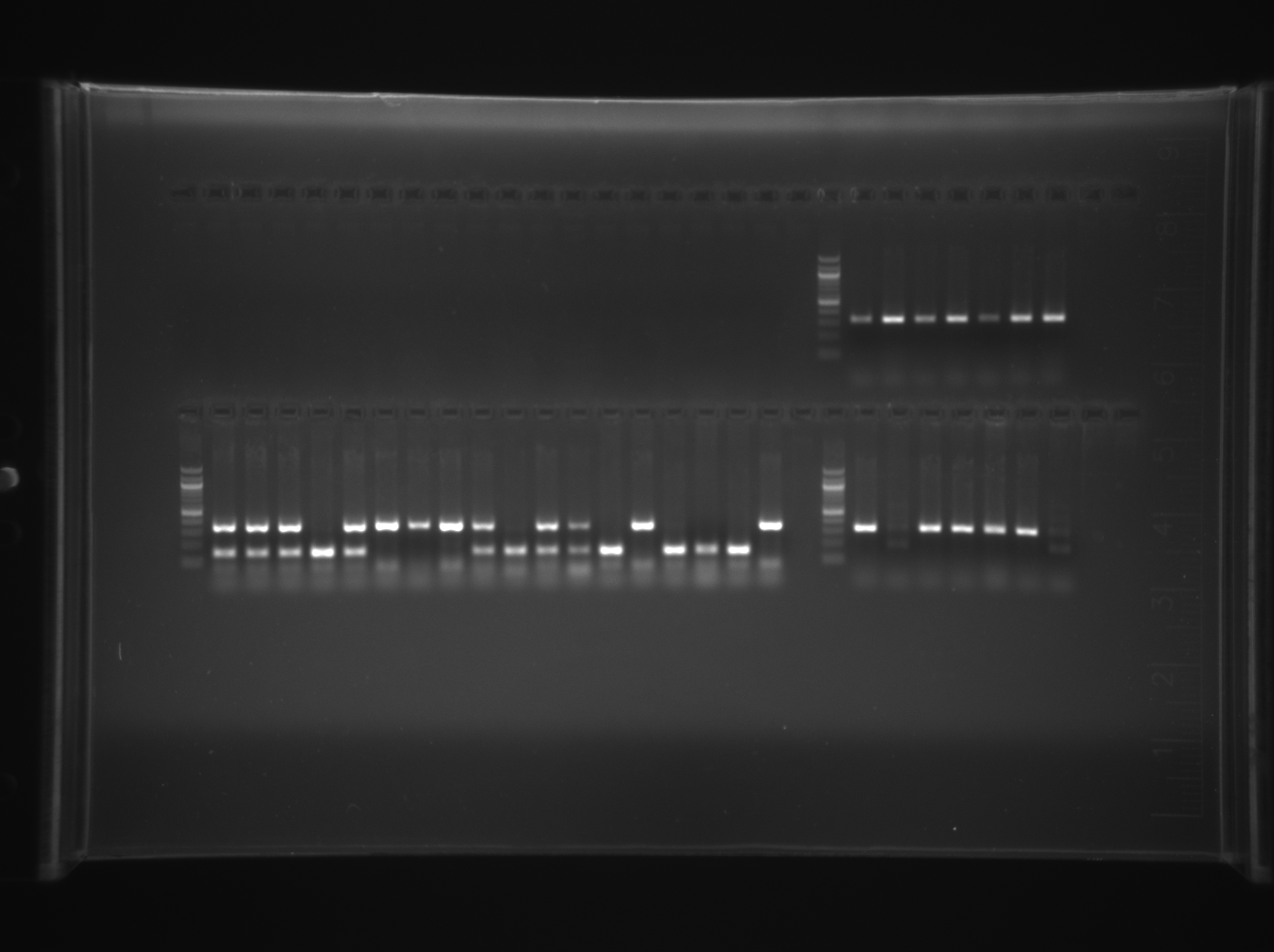

Supplement: Original Image for Fig 1A.tif [file KGMR_A_2548210_SM4050.tif]

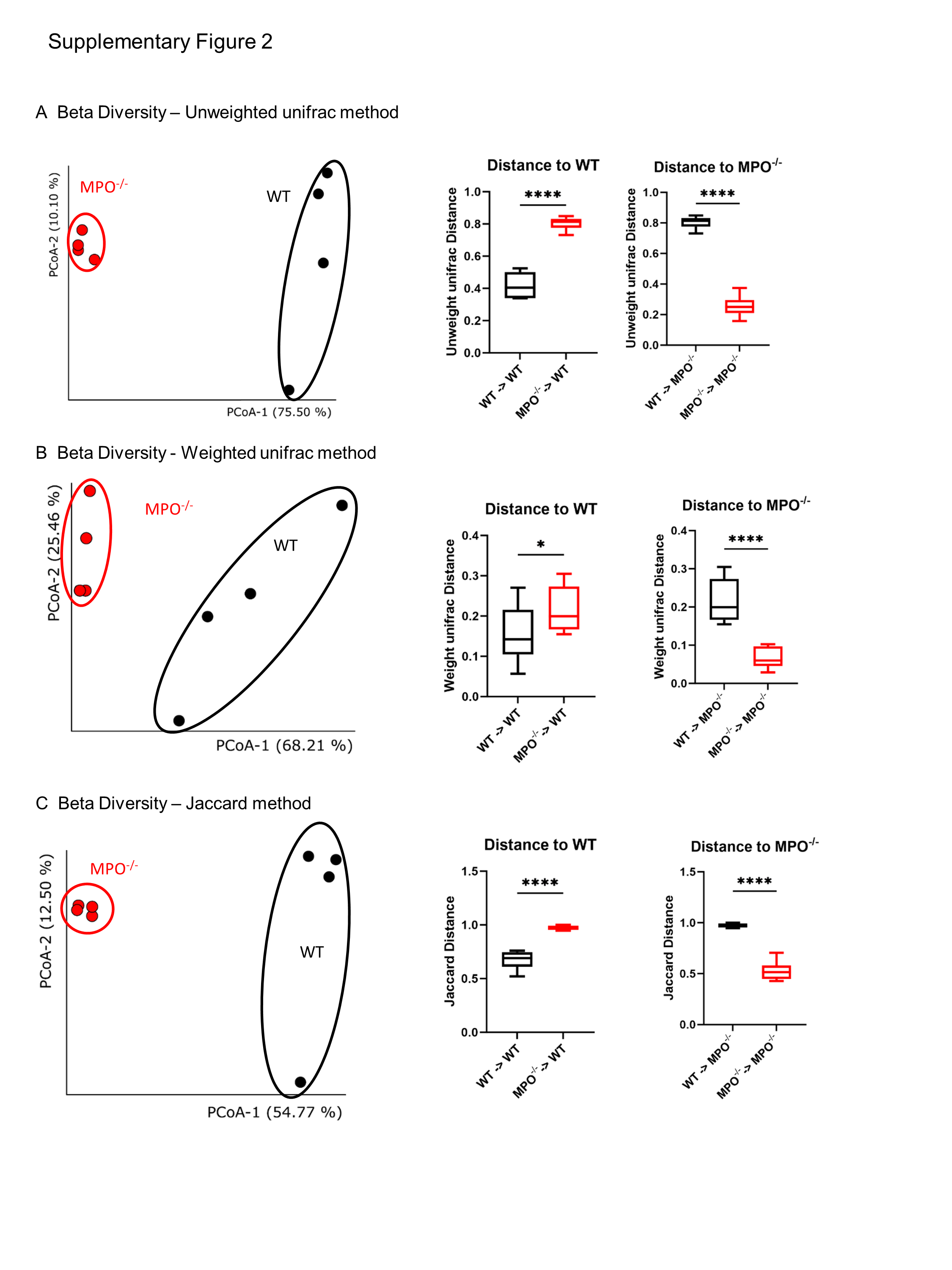

Supplement: Figure S2.TIF [file KGMR_A_2548210_SM4049.tif]
