# Supplementary material for: Myeloperoxidase gene knockout causes local inflammation and dysbiosis in the murine gut
Source: Gut Microbes Rep. 2025 Aug 28;2(1):2548210. doi: 10.1080/29933935.2025.2548210 (PMC12700598; doi:10.1080/29933935.2025.2548210)
Supplement: Wang et al 2025_Supplementary Information.docx [file KGMR_A_2548210_SM4048.docx]

**Supplementary Data**

**Myeloperoxidase Loss of Function Leads to Intestinal Inflammation and Dysbiosis**

Jack J. Wang, Yawen Hu, Scott Jennings, Meng Luo, Christopher M. Taylor,

William M. Nauseef, Guoshun Wang

Figure S1. Gating strategy of flow cytometry.

Figure S2. Beta diversity of bacterial community in the gut of WT and MPO^-/-^ mice by other methods.

Table S1. Immunostaining pane

Table S2. Information of Raw FASTQ Data Quality Control

Table S3. Results of diversity analysis

Table S4. Taxonomy and differential abundance analysis of bacteria at family level and genus level

Table S5. Pathway abundance

Table S6. Differential analysis of pathway abundance

**Figure S1**

**Fig. S1.** Gating strategy of flow cytometry.

**Figure S2**

**Fig. S2.** Beta diversity of bacterial community in the gut of WT and MPO^-/-^ mice by other methods. (A) Unweighted unifrac method. (B) Weighted unifrac method. (C) Jaccard method. Significant differences were determined by a 2-tailed, unpaired Student’s t-test. *p<0.05, ***p<0.001, ****p<0.0001.

**Table S1**

| Name | Fluorophore | Company | Catalog No. | Lot No. | Clone # | Dilution |
| --- | --- | --- | --- | --- | --- | --- |
| Rat anti-mouse CD45 | BUV395 | BD Horizon | 564279 | 2004510 | 30-F11 | 1:200 |
| Rat anti-CD11b | FITC | BM Pharmingen | 561688 | 9162746 | M1/70 | 1:200 |
| Rat anti-mouse F4/80 | APC | BM Pharmingen | 566787 | 1291912 | T45-2342 | 1:200 |
| Rat anti-mouse CD19 | BV711 | BD Horizon | 563157 | 1222281 | 1D3 | 1:200 |
| Hamster anti-mouse CD3ε | PE | BM Pharmingen | 561824 | 2026326 | 145-2C11 | 1:200 |
| Rat anti-mouse Ly6C | BV605 | BD Horizon | 563011 | 1293081 | AL-21 | 1:200 |
| Rat anti-mouse Ly6G | BV421 | BD Horizon | 562737 | 3132029 | 1A8 | 1:200 |
| Purified rat anti-mouse CD16/CD32 (Mouse BD Fc blocker) |  | BD Pharmingen | 553142 | 1293775 | 2.4G2 | 1:100 |
| Fixable Viability Stain 780 |  | BD Horizon | 565388 |  |  | 1:500 |
| Brilliant Stain Buffer Plus |  | BD Horizon | 566385 | 2004540 |  | 10µl/test |

**Tables S2-S6**

See excel files
